# Inhibition of *Mycoplasma pneumoniae* growth by FDA-approved anticancer and antiviral nucleoside and nucleobase analogs

**DOI:** 10.1186/1471-2180-13-184

**Published:** 2013-08-06

**Authors:** Ren Sun, Liya Wang

**Affiliations:** 1 Department of Anatomy, Physiology, and Biochemistry, Swedish University of Agricultural Sciences, The Biomedical Centre, Box 575, SE-751 23 Uppsala, Sweden

**Keywords:** *Mycoplasma pneumoniae*, Growth inhibition, 6-thioguanine, Trifluorothymidine, Hypoxanthine guanine phosphoribosyl transferase, Thymidine kinase

## Abstract

**Background:**

*Mycoplasma pneumoniae* (Mpn) is a human pathogen that causes acute and chronic respiratory diseases and has been linked to many extrapulmonary diseases. Due to the lack of cell wall, Mpn is resistant to antibiotics targeting cell wall synthesis such as penicillin. During the last 10 years macrolide-resistant Mpn strains have been frequently reported in Asian countries and have been spreading to Europe and the United States. Therefore, new antibiotics are needed. In this study, 30 FDA-approved anticancer or antiviral drugs were screened for inhibitory effects on Mpn growth and selected analogs were further characterized by inhibition of target enzymes and metabolism of radiolabeled substrates.

**Results:**

Sixteen drugs showed varying inhibitory effects and seven showed strong inhibition of Mpn growth. The anticancer drug 6-thioguanine had a MIC (minimum inhibitory concentration required to cause 90% of growth inhibition) value of 0.20 μg ml^-1^, whereas trifluorothymidine, gemcitabine and dipyridamole had MIC values of approximately 2 μg ml^-1^. In wild type Mpn culture the presence of 6-thioguanine and dipyridamole strongly inhibited the uptake and metabolism of hypoxanthine and guanine while gemcitabine inhibited the uptake and metabolism of all nucleobases and thymidine. Trifluorothymidine and 5-fluorodeoxyuridine, however, stimulated the uptake and incorporation of radiolabeled thymidine and this stimulation was due to induction of thymidine kinase activity. Furthermore, Mpn hypoxanthine guanine phosphoribosyl transferase (HPRT) was cloned, expressed, and characterized. The 6-thioguanine, but not other purine analogs, strongly inhibited HPRT, which may in part explain the observed growth inhibition. Trifluorothymidine and 5-fluorodeoxyuridine were shown to be good substrates and inhibitors for thymidine kinase from human and *Mycoplasma* sources.

**Conclusion:**

We have shown that several anticancer and antiviral nucleoside and nucleobase analogs are potent inhibitors of Mpn growth and that the mechanism of inhibition are most likely due to inhibition of enzymes in the nucleotide biosynthesis pathway and nucleoside transporter. Our results suggest that enzymes in *Mycoplasma* nucleotide biosynthesis are potential targets for future design of antibiotics against *Mycoplasma* infection.

## Background

*Mycoplasmas* are wall-less, gram-positive bacteria and are pathogenic to humans, animals, and plants [1]. *Mycoplasma pneumoniae* (Mpn) is a human pathogen and causes acute and chronic diseases at multiple sites. Respiratory diseases dominate and account for approximately 30% of cases of community-acquired pneumonia. Mpn may also be a direct cause or significant cofactor in many extrapulmonary diseases including rheumatoid arthritis, and central nerve system manifestations such as encephalitis, aseptic meningitis, acute transverse myelitis, stroke, chronic fatigue, and polyradiculopathy [1-3]. Due to the lack of cell wall, Mpn is resistant to antibiotics targeting cell wall synthesis such as penicillin, and macrolides are the treatment of choice.

Increased incidences/epidemics of Mpn infections have been reported in Scandinavian countries, France, Scotland, and Israel from 2010 to 2012 [4,5]. In most cases, the infected individuals did not need medical attention. However, approximately 10% of the patients developed pneumonia and antibiotic treatment was needed. In severe cases, hospitalization was required and there were lethal cases when patients were infected by macrolide-resistant Mpn strains [6,7]. During the last 10 years macrolide-resistant Mpn strains have been frequently reported in Asian countries and have been spreading to Europe and the United States. In Japan and China, approximately 90% of the isolates are resistant to erythromycin or azithromycin, especially among pediatric patients [8-12]. This limits treatment options for patients with severe *Mycoplasma* pneumonia caused by macrolide resistant Mpn strains. Therefore, new antibiotics are needed.

Nucleotides are not only the building blocks of DNA and RNA, but also regulatory factors in diverse cellular metabolic pathways, and therefore, inhibition of enzymes in this pathway will cause nucleotide pool imbalance, which will inhibit DNA and RNA synthesis and lead to cell death. When transported into and metabolized by cells, nucleoside analogs can interfere with metabolism of natural nucleotides and/or DNA and RNA synthesis. An example of this type of antibiotic is sulphonamides such as sulfamethoxazole that target dihydropteroate synthetase in the folic acid biosynthesis pathway, and inhibition of folic acid biosynthesis leads to impaired purine and pyrimidine nucleotide biosynthesis [13]. Another example is thymidylate synthase (TS) inhibitors such as Ralitrexed and 5-fluorouracil used as anticancer drugs [14,15]. Today more than 50% of the United States Food and Drug Administration (FDA)-approved anticancer and antiviral drugs are nucleoside and nucleobase analogs. The most successful reports since the 1970s, using nucleoside analogs as drugs, were for the treatment of herpes viral infections by acyclic guanosine analogs such as acyclovir, and HIV infection by nucleoside analogs such as Zidovudine or Lamivudine in combination with protease inhibitors i.e., highly active antiretroviral therapy [16,17].

Compared to other antibacterial compounds, most nucleoside and nucleobase analogs used in anticancer and antiviral therapy have narrow therapeutic index and adverse side effects, with the exception of acyclic guanosine analogs used in the treatment of herpes viral infection. These adverse effects limit their use in the treatment of bacterial infections. In recent years efforts have been made to develop nucleoside analog based antibiotics, taking the advantage of structural differences in target enzymes between bacteria and their host to design and select specific inhibitors that will selectively kill the bacteria and spare the host [18-23]. For example, selective thymidylate kinase inhibitors have been developed and showed potent inhibitory effect *in vivo* against methicillin-resistant *Staphylococcus aureus* and vancomycin-resistant *Enterococcus*[22,23]. Toxicity or side effect of these thymidylate kinase inhibitors to humans remains to be seen.

*Mycoplasmas*, in general, depend on exogenous supply of precursors for their nucleotide biosynthesis because they lack the *de novo* synthesis of purine and pyrimidine bases. Nucleosides and deoxynucleosides are efficiently taken up and phosphorylated to their respective nucleotides by deoxynucleoside kinases such as thymidine kinase (TK) and deoxyadenosine kinase. Nucleobases are salvaged through hypoxanthine guanine phosphoribosyltransferase (HPRT), adenine phosphoribosyltransferase (APRT) and uracil phosphoribosyltransferase (UPRT) systems [24-32]. Of a total of 17 enzymes in nucleotide biosynthesis identified in the Mpn genome, 15 are essential. Enzymes mentioned above, TK, HPRT, APRT and UPRT are essential for Mpn survival while thymidylate synthase (TS), an enzyme catalyses the reductive synthesis of thymidylate from uridylate, is not since *thyA* mutant Mpn strain that lacks TS is viable [31,33,34].

In this study, 30 FDA-approved nucleoside and nucleobase analogs that are anticancer or antiviral drugs were screened for inhibitory effects on Mpn growth. Seven analogs showed potent inhibitory effects on Mpn growth at clinically achievable plasma concentrations. Among them, 6-thioguanine (6-GT) inhibited Mpn growth with a MIC (minimum inhibitory concentration required to cause 90% of growth inhibition) value of 0.20 μg ml^-1^. To investigate the mechanism of action of these drugs, we studied the effects of these analogs on uptake and metabolism of natural nucleoside and nucleobases by using tritium labelled natural substrates. Furthermore Mpn hypoxanthine guanine phosphoribosyl transferase (HPRT) was cloned and expressed, and the recombinant enzyme was purified and characterized using tritium labelled hypoxanthine and guanine as substrates, and 6-thiuoguanine and other purine analogs as inhibitors. The role of thymidine kinase in the inhibitory effect of trifluorothymidine against Mpn growth was also investigated.

## Results

### Inhibition of Mpn growth by nucleoside and nucleobase analogs

Some nucleoside analogs have been reported to inhibit *Mycoplasma* growth [30,35]. Recently a nucleoside and nucleobase analog library consisting of FDA-approved prodrugs used in anticancer and antiviral therapy was used to screen human enzymes in nucleotide metabolism, and new interactions were found [36], which promoted the use of these analogs in screening for inhibitory effects on Mpn growth. A series dilution of each compound in *Mycoplasma* growth medium was added to Mpn cultures in 96-well plates. The plates were sealed and incubated at 37°C. Mpn growth was monitored by using growth index value e.g. the ratio of absorbance at 450 nm and 560 nm of the culture medium [32]. Thirty nucleoside and nucleobase analogs and a nucleoside transporter inhibitor were included, and two Mpn strains, wild type and a *thyA* mutant (lacking TS activity), were used. Sixteen of these compounds inhibited Mpn growth to varying levels, and seven showed strong inhibition (Table 1). The anticancer drug 6-TG and the antiviral and anticancer drug trifluorothymidine (TFT) strongly inhibited Mpn growth, with MIC values of 0.2 μg ml^-1^ and 1.8 μg ml^-1^, respectively. Gemcitabine (dFdC), an anticancer agent, was also strong inhibitor of Mpn growth with MIC of approximately 2.5 μg ml^-1^. Dipyridamole, a nucleoside transporter inhibitor, also strongly inhibited Mpn growth with MIC of 1.9 μg ml^-1^ (Table 1). All analogs had MIC values at clinically achievable plasma concentrations. The cultures were kept for additional 3 weeks in the incubator and there was no indication of growth.

**Table 1:** Inhibition of *M. pneumoniae *growth by nucleoside and nucleobase analogs*

**Compounds**	**Wild type MIC (μg ml**^**-1**^**)**	***thyA*mutant MIC (μg ml**^**-1**^**)**
Ribavirin	62.5	> 500
Pentoxifylline	62.5	> 500
Gancyclovir	7.8	> 500
Zidovudine	7.8	7.8
Gemcitabine (dFdC)	2.4	2.4
Stavudine	7.8	17.8
Acyclovir	15.6	15.6
Pyrimethamine	> 500	> 500
Fludarabine phosphate	> 500	> 500
Lamivudine	> 500	> 500
Mycophenolate mofetil	250	250
Trifluorothymidine (TFT)	1.8	1.8
Adefovir depivoxil	> 500	> 500
5-azacytidine	> 500	> 500
Azathioprine	> 500	> 500
Arabinosyl adenine	> 500	> 500
Zalcitabine	> 500	> 500
5-iododeoxyuridine	15.6	> 500
5-fluorodeoxyuridine (5FdU)	7.8	15.6
Cidofovir	31.2	31.2
Caffeine	> 500	> 500
7-(2,3-dihydroxypropyl)theophylline	> 500	> 500
Theophylline	> 500	> 500
6-thioguanine (6-TG)	0.2	0.2
Allopurinol	> 500	> 500
6-mercaptopurine (6-MP)	> 500	> 500
5-fluorouracil	31.2	31.2
5-fluorocytosine	31.2	31.2
Valacyclovir	> 500	> 500
Dipyridamole	1.9	1.9

*MIC = minimal concentrations of the compound that produced 90% inhibition.

For most compounds, the inhibitory effects were similar between the wild type and the *thyA* mutant Mpn strains, however differences between the two Mpn strains were also observed. For example, gancyclovir inhibited wild type Mpn but not the *thyA* mutant, whereas valacyclovir did not inhibit Mpn growth. Ribavirin and pentoxifylline inhibited wild type Mpn but not the *thyA* mutant. Among the 5-halogenated pyrimidine analogs, most of them inhibited both the wild type and the *thyA* mutant strain, but 5-iododeoxyuridine only inhibited the wild type Mpn growth (Table 1)*.*

### Uptake and metabolism of natural nucleosides and nucleobases in the presence of analogs

To investigate the mechanism of inhibition by these analogs, we incubated Mpn wild type cells with radiolabelled natural substrates in the presence and absence of those analogs that strongly inhibited Mpn growth. Total uptake and metabolism of the radiolabelled substrates were analysed to access whether the observed growth inhibition is at the level of nucleoside transporter or metabolism or both (Table 2). In general the uptake of nucleobases e.g. [^3^H]-Ade (adenine), [^3^H]-Gua (guanine), [^3^H]-Ura (uracil), and [^3^H]-Hx (hypoxanthine) was low (< 1%) as compared with that of [^3^H]-dT (thymidine) (> 7%). Dipyridamole strongly inhibited the uptake and incorporation of [^3^H]-Hx and [^3^H]-Gua into DNA and RNA but had no effect on uptake and metabolism of all other nucleobases and [^3^H]-dT, suggesting that dipyridamole is a specific inhibitor of purine transport. Similar to dipyridamole, 6-TG also strongly inhibited the uptake and incorporation of [^3^H]-Hx and [^3^H]-Gua into DNA and RNA but had no effect on any other nucleobases and dT. Pyrimidine nucleoside analogs, TFT, 5FdU (5-fluorodeoxyuridine) and dFdC, inhibited the uptake and incorporation of all nucleobases. However, [^3^H]-dT uptake was stimulated (2-fold) by TFT and 5FdU but inhibited by dFdC, and the percentage of radioactivity found in DNA was similar to that of control in all cases (Table 2). These results indicate that there are distinct transporters for purines and pyrimidines and that metabolic rate determines the extent of uptake.

**Table 2:** Inhibition of tritium labelled natural nucleoside and nucleobase uptake and metabolism by selected analogs*

	**[**^**3**^**H]-dT**		**[**^**3**^**H]-Ura**		**[**^**3**^**H]-Hx**		**[**^**3**^**H]-Gua**		**[**^**3**^**H]-Ade**	
	**Total uptake**	**Incorporation**	**Total uptake**	**Incorporation**	**Total uptake**	**Incorporation**	**Total uptake**	**Incorporation**	**Total uptake**	**Incorporation**
None	7.6±0.5	97.5±0.5	0.20±0.003	40±5	0.050± 0.001	62±7	0.9±0.05	56±3	0.62±0.1	44±1
Dipyridamole	7.2±1.1	97.0±1.3	0.20±0.003	38±6	0.008± 0.001	44±3	0.09±0.002	56±6	0.67±0.1	47±1
6-TG	7.9±0.6	97.4±0.7	0.21±0.003	39±8	0.005 ± 0.0004	43±6	0.080±0.002	67±3	0.66±0.1	46±3
TFT	18.2±0.6	97.4±0.5	0.11±0.002	27±0.2	0.011± 0.001	67±1	0.19±0.02	85±4	0.43±0.01	48±2
5FdU	14.7±0.2	96.0±0.5	0.087±0.003	19±7	0.006± 0.001	76±4	0.16±0.03	87±3	0.36±0.1	42±2
dFdC	5.2±0.4	96.7±1.1	0.12±0.001	26±6	0.009±0.0002	67±7	0.10±0.02	90±6	0.41±0.08	39±8

*Total uptake: percentage of radioactivity recovered in the cells divided by total radioactivity added to the growth medium.

Incorporation: percentage of radioactivity in the acid insoluble fraction divided by total radioactivity recovered in the cells.

### Up-regulation of Mpn TK activity by TFT

**Figure 1:**
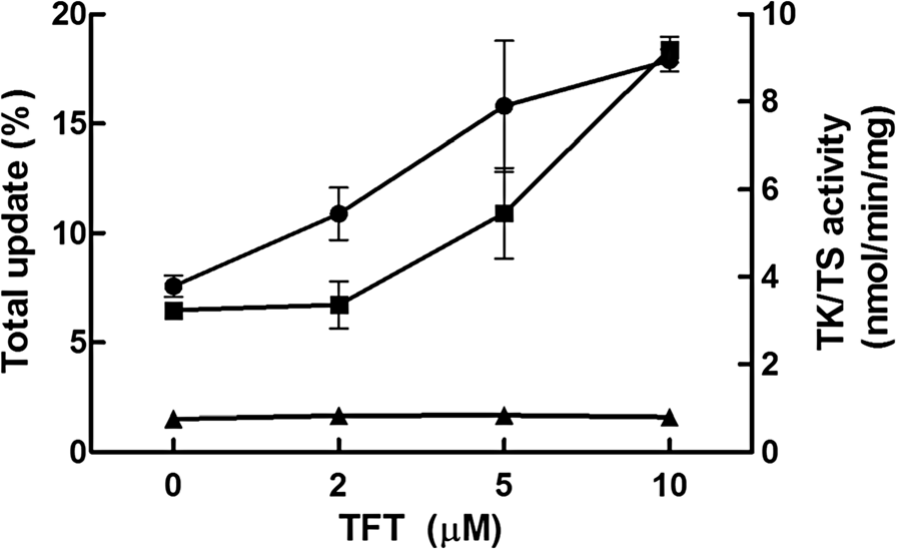
**The effect of trifluorothymidine (TFT) on the uptake of [**^**3 **^**H]-dT (●), TK (■) and TS (▲) activity.** Mpn wild type cells were cultured in the presence of [^3^H]-dT and different concentrations of TFT. The cells were incubated at 37°C for 70 hours and harvested. The total uptake and incorporation of [^3^H]-dT were analysed, and TK and TS activity were determined in total protein extracts.

To understand why TFT and 5FdU stimulated [^3^H]-dT uptake, Mpn wild type cells were incubated with various concentrations of TFT in the presence of [^3^H]-dT. Total proteins were extracted from these cultures and used to determine the TK and TS activity. Total uptake of [^3^H]-dT increased in a concentration dependent manner while the percentage of [^3^H]-dT found in DNA was similar. TK activity increased also as the concentration of TFT increases and with 10 μM TFT the TK activity was ~ 3 times of the activity found in the controls. TS activity, however, was unchanged (Figure 1). These results demonstrated that TFT induced up-regulation of TK activity but had no effect on TS activity, and suggested that the high level of dT uptake in the presence of TFT and 5FdU is due to increased TK activity.

### Expression, purification, and characterization of HPRT

The purine analog 6-TG strongly inhibited Mpn growth, which promoted further investigation of potential targets of this compound. HPRT is the first enzyme in the salvage pathway of purine bases for nucleotide biosynthesis, and is the enzyme responsible for metabolizing 6-TG in human patients treated with this drug [37]. Mpn HPRT (MPN672) consists of 175 amino acids and shares 29% sequence identity to human HPRT. Mpn HPRT cDNA was cloned and expressed in *E. coli.* Recombinant Mpn HPRT was expressed as an N-terminal fusion protein with a 6 × histidine tag and a tobacco etch virus (TEV) cleavage site at the N-terminus, and was purified to >98% purity by metal affinity chromatography, as assessed by sodium dodecyl sulfate polyacrylamide gel electrophoresis (SDS-PAGE) analysis (data not shown).

**Figure 2:**
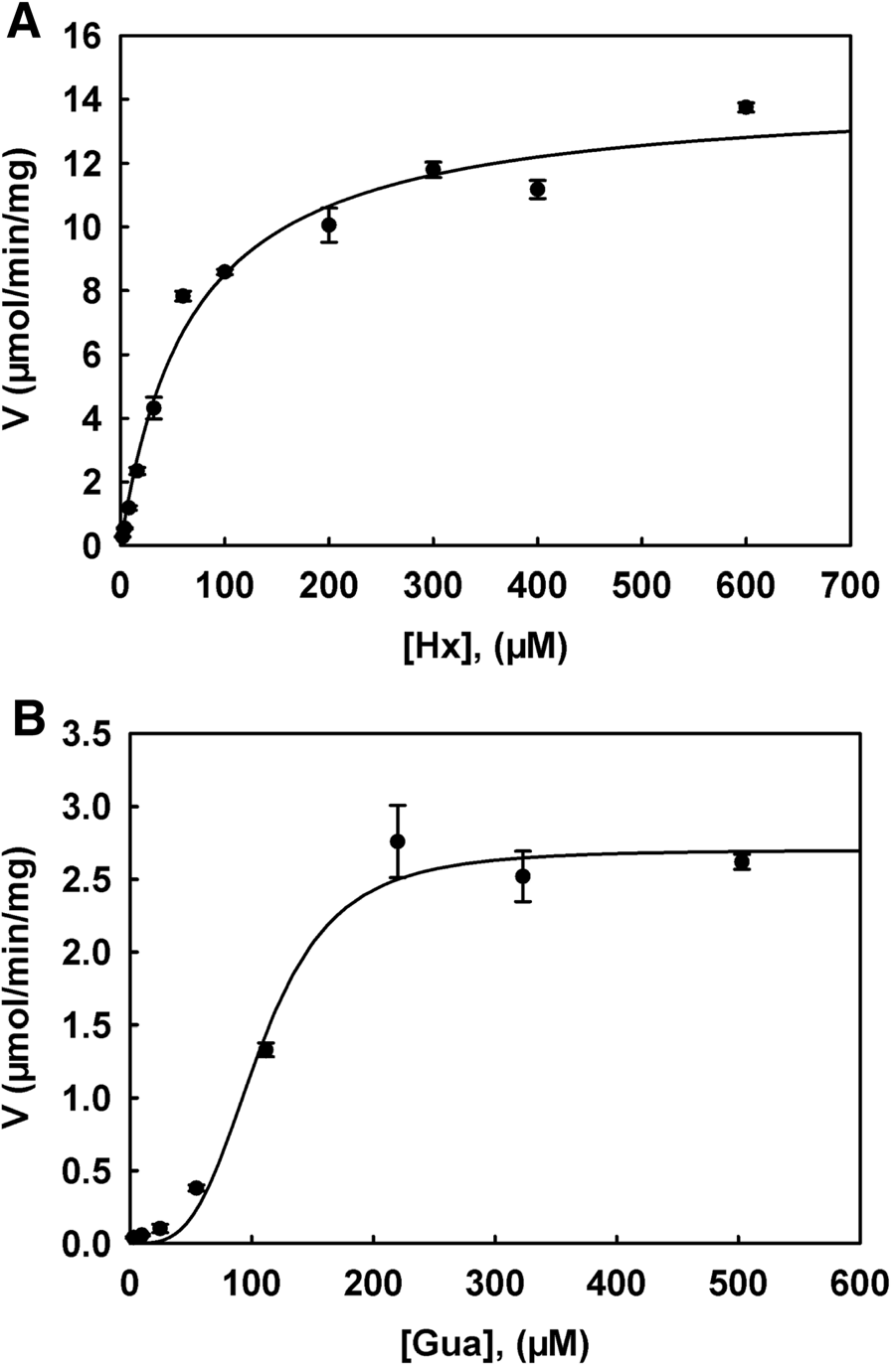
**Substrate saturation curves of hypoxanthine (A) and guanine (B) with Mpn HPRT.** Kinetic parameters for Hx and Gua were determined by using the DE81 filter paper assay with [^3^H]-Hx and [^3^H]-Gua as the labelled substrates as described in the experimental procedures. Data are from at least three independent measurements and are presented as mean ± standard deviation (SD).

The purified Mpn HPRT used both hypoxanthine (Hx) and guanine (Gua) as substrates but not adenine or uracil. With Hx as substrate the reaction was linear with time for up to 25 min and the substrate saturation curve was hyperbolic, which indicated that the enzyme followed Michaelis–Menten kinetics with a K_m_ value of 100.1 ± 6.5 μM and V_max_ value of 15.8 ± 0.8 μmol min^-1^ mg^-1^ (Figure 2A). However, with Gua as a substrate, the reverse reaction rate was very high and the reaction reached equilibrium in less than 5 min under the same conditions used for Hx. Therefore, the kinetic study with Gua was conducted differently as described in the experimental procedures. Substrate saturation for Gua exhibited a biphasic curve and therefore, data was fitted using the Hill equation. The V_max_ value was 2.7 ± 0.1 μmol min^-1^ mg^-1^ and S_0.5_ was 107.6 ± 6.2 μM with a Hill coefficient of 3.5 (Figure 2B), indicating positive cooperativity with Gua binding.

### Inhibition of Mpn and human HPRT with 6-TG and other purine analogs

To study the mechanism of inhibition by 6-TG on Mpn growth, we determined the inhibitory effect of 6-TG, 6-mercaptopurine (6-MP), and two other purine analogs that did not inhibit Mpn growth on Mpn HPRT, and compared the results with human HPRT. The 6-TG strongly inhibited Mpn HPRT with either Hx or Gua as a substrate, and the half maximal inhibitory concentration (IC_50_) values were 3.5 ± 0.5 μM (Hx) and 3.2 ± 0.2 μM (Gua). The 6-TG also inhibited human HPRT, but with a much higher IC_50_ value (Table 3). The 6-MP inhibited Mpn HPRT with IC_50_ values of 89.7 ± 14.5 μM (Hx) and 281.9 ± 21 μM (Gua). With human HPRT, 6-MP had similar IC_50_ values to those of 6-TG. Theophylline and caffeine were poor inhibitors of both Mpn and human HPRT (Table 3).

**Table 3:** Inhibition of Mpn and human HPRT by purine analogs^*^

**Substrate**	**Hypoxanthine**			**Guanine**		
**Analogs**	**IC**_**50**_**(μM)**			**IC**_**50**_**(μM)**		
	**Mpn**	**Human**	**P value**	**Mpn**	**Human**	**P value**
6-thioguanine	3.5 ± 0.5	20.5 ± 6.5	0.0107	3.16 ± 0.2	39.8 ± 4	<0.0001
6-mercaptopurine	89.7 ± 14.5	22.5 ± 3.6	0.0015	281.8 ± 21	25.1 ± 3	<0.0001
Theophylline	> 4000	1585 ± 134		nd	nd	
Caffeine	> 4000	2511 ± 156		nd	nd	

*Assays were performed with 10 μM tritium labelled hypoxanthine or guanine as substrates and various concentrations of the inhibitors. Data were mean ± standard deviation (SD) from at least three independent determinations. P < 0.05 is considered as significant.

nd = not determined.

### Trifluorothymidine (TFT) as substrate for human thymidine kinase (TK) 1, TK2, and *Ureaplasma* TK

**Figure 3:**
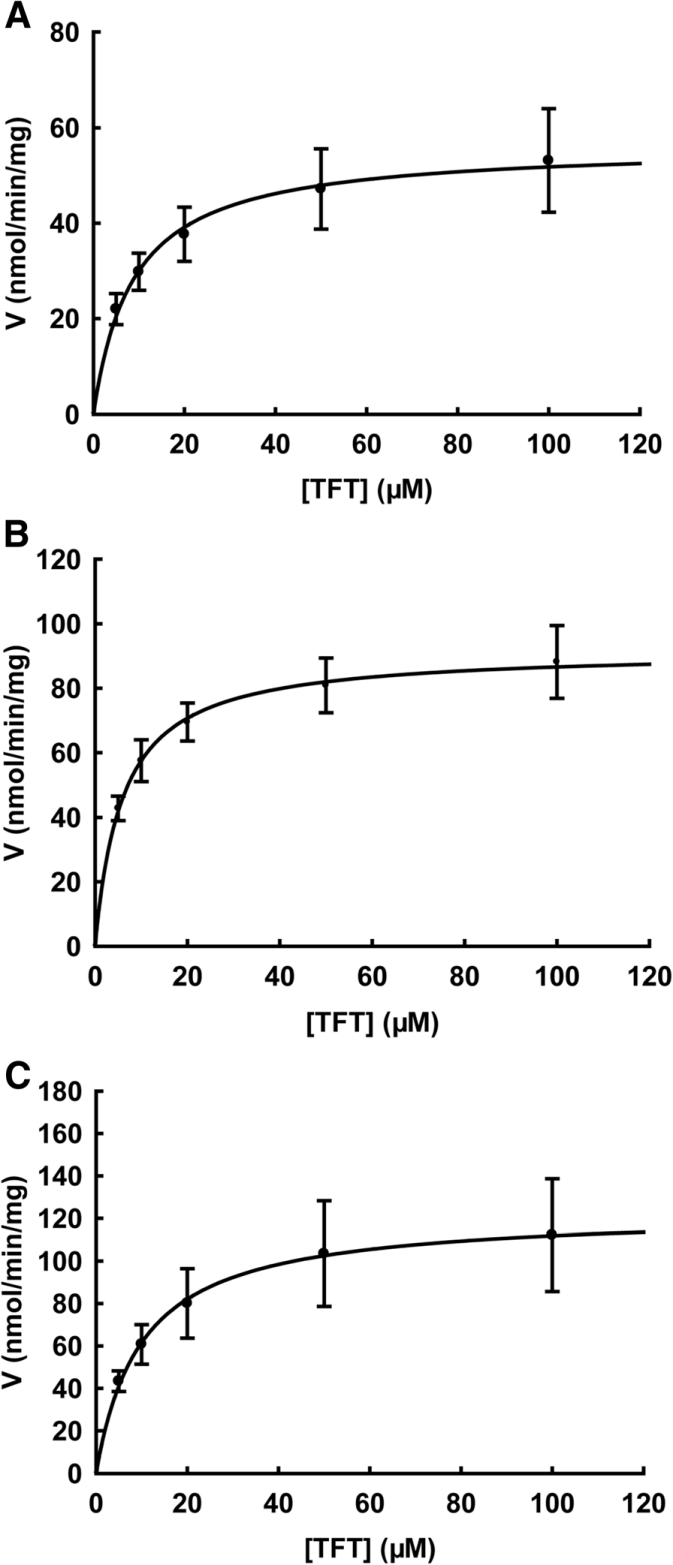
**Substrate saturation curves of TFT with human TK2 (A), human TK1 (B), and *Ureaplasma *TK (C).** Kinetic assays with TFT were performed by using [γ-^32^P]-ATP as the labelled substrate at fixed concentration and variable concentrations of TFT. The reaction products were separated by thin layer chromatography, and quantified as described in the experimental procedures. Data are from three independent measurements and are presented as mean ± SD.

TFT is a known substrate of human TK1, and has been used as an antiviral and anticancer drug. We found that TFT strongly inhibited Mpn growth, suggesting that Mpn TK may have a role in growth inhibition caused by TFT. The mechanism of inhibition by TFT and 5-fluorodeoxyuridine (5FdU) was proposed to be linked to inhibition of TS [38]. However, we observed that TFT and 5FdU inhibited both the wild type and the *thyA* mutant strains to similar extents, suggesting that the mechanism of inhibition is unlikely to be solely by inhibition of TS activity. Therefore, we sought to characterize TFT phosphorylation by *Mycoplasma* TK and compared this with the human enzymes. Kinetic studies with TFT were performed with purified recombinant human TK1 (a cytosolic enzyme), TK2 (a mitochondrial isoenzyme), and *Ureaplasma* TK. Because *Ureaplasma* TK and Mpn TK share 46% sequence identity and they also share 36% respective 32% sequence identity to human TK1 [30,39], and Mpn TK is not available. The phosphorylation of TFT by human TK1, TK2, and *Ureaplasma* TK followed Michaelis-Menten kinetics and the K_m_ values for the three enzymes were in the same range, while the k_cat_ values varied between the three enzymes (Figure 3). *Ureaplasma* TK had the highest k_cat_ value and human TK2 had the lowest k_cat_ value (Table 4). However, the overall efficiency was highest with human TK1 and lowest with human TK2 (Table 4).

**Table 4:** Kinetic parameters of trifluorothymidine with purified recombinant human TK1, TK2, and *Ureaplasma *TK*

	**K**_**m**_**(μM)**	**k**_**cat**_**(s**^**-1**^**)**	**k**_**cat**_**/K**_**m**_**(s**^**-1**^**M**^**-1**^**)×10**^**3**^
Human TK1	5.9 ± 1.7	0.043 ± 0.003	7.3 ± 1.8
Human TK2	8.8 ± 3.8	0.026 ± 0.003	3.0 ± 0.8
*Ureaplasma* TK	9.9 ± 5.2	0.055 ± 0.008	5.6 ± 1.5

*Assays were performed using phosphoryl transfer assay with [γ-^32^P]-labelled ATP (100 μM) and variable concentrations of TFT (1 – 100 μM). The reaction products were separated by thin layer chromatography and were quantified. Thymidine (10 μM) was used as a control. Data are from three independent measurements and are expressed as mean ± SD.

### Inhibition of human TK1, TK2, and *Ureaplasma* and Mpn TK by TFT and 5FdU

Both TFT and 5FdU are substrates of *Mycoplasma* and human TKs, as described above and earlier studies [30,40,41]. However, their inhibitory effects on these enzymes are not known, and inhibition of TK activity by these two analogs may account for the observed Mpn growth inhibition. Therefore, we determined the IC_50_ values for TFT and 5FdU with dT as a substrate and found significant differences in IC_50_ values between TFT and 5FdU for all enzymes. TFT inhibited dT phosphorylation in Mpn protein extracts with an IC_50_ value of 9.1 ± 2.9 μM, which was similar to that of recombinant *Ureaplasma* TK. With recombinant human TK1 and TK2, the IC_50_ values were 9.7 ± 3.2 μM and 80 ± 5.6 μM, respectively. The inhibition by 5FdU was much weaker for all recombinant enzymes and Mpn extracts (Table 5). Thus, TFT was a significantly better inhibitor than 5FdU.

**Table 5:** **IC**_**50 **_values (μM) of trifluorothymidine (TFT) and 5-fluorodeoxyuridine (5FdU) with purified recombinant human TK1 and TK2, *Ureaplasma *TK, and Mpn extracts^*^

	**TFT**	**5FdU**	**P value**
Human TK1	9.7 ± 3.2	75.9 ± 2.6	<0.0001
Human TK2	80 ± 5.6	158.5 ± 2.7	<0.0001
*Ureaplasma* TK	12.0 ± 4.2	1000 ± 13.3	<0.0001
Mpn extracts	9.1 ± 2.9	47.9 ±1.2	<0.0001

^*^Assays were performed with 10 μM tritium labelled thymidine as substrate in the presence of various concentrations of the inhibitors. Data were mean ± SD from at least three independent determinations. P value < 0.05 is considered as significant.

## Discussion

**Figure 4:**
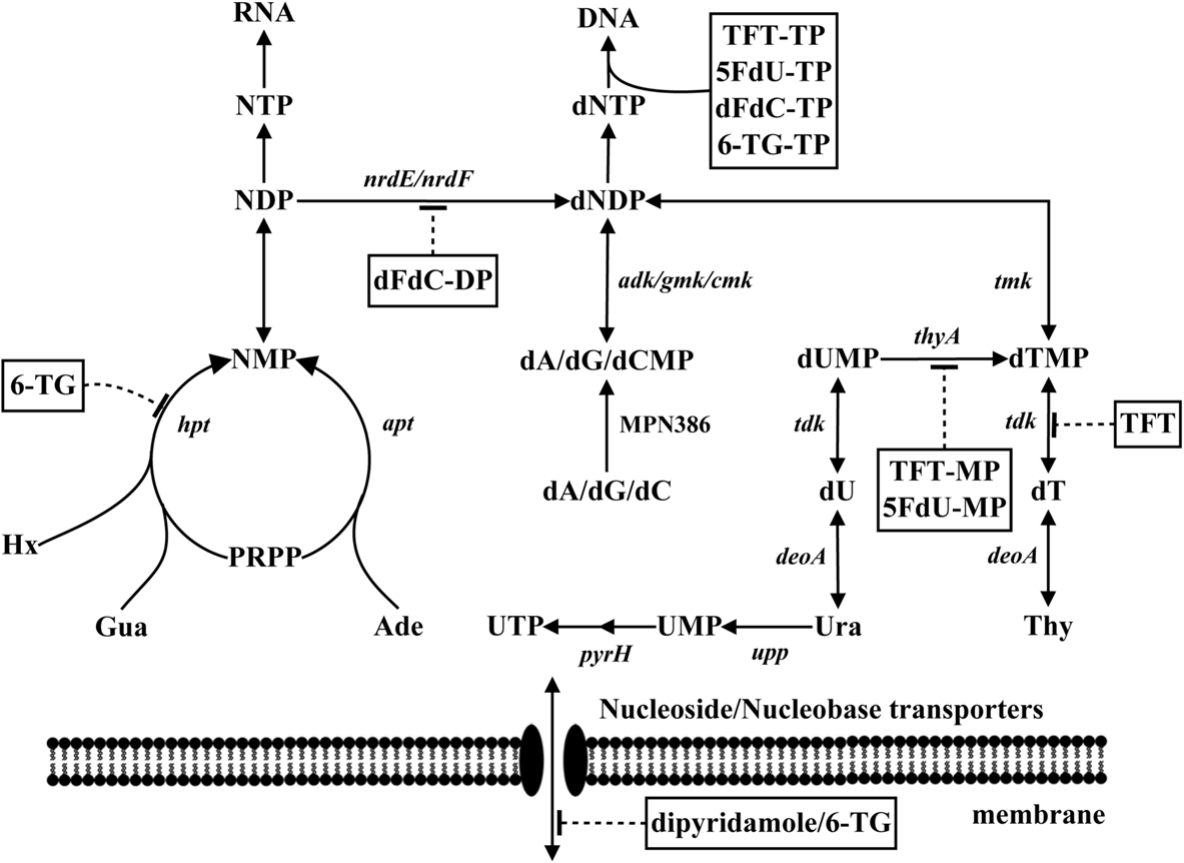
**Schematic overview of *M. pneumoniae *nucleotide biosynthesis*.*** Hx, hypoxanthine; Gua, guanine; Ura, uracil; Thy, thymine; dT, thymidine; dA, deoxyadenosine; dC deoxycytidine; dG, deoxyguanosine; PRPP, phosphoribosyl pyrophosphate; NMP, nucleoside monophosphate; NDP, nucleoside diphosphate, NTP, nucleoside triphosphate; dNDP, deoxynucleoside diphosphate; dNTP, deoxynucleoside triphosphate; TFT, trifluorothymidine; TFT-MP, trifluorothymidine monophosphate; TFT-TP, trifluorothymidine triphosphate; 5FdU-MP, 5-fluorodeoxyuridine monophosphate; 5FdU-TP, 5-fluorodeoxyuridine triphosphate; dFdC-DP, gemcitabine diphosphate; dFdC-TP, gemcitabine triphosphate; 6-TG, 6-thioguanine; 6-TG-TP, 6-thioguanine triphosphate. Enzymes: *hpt*, hypoxanthine guanine phosphoribosyl transferase (MPN672); *apt*, adenine phosphoribosyl transferase (MPN395); *upp*, uracil phosphoribosyl transferase (MPN033); *deoA*, thymidine phosphorylase (MPN064); *tdk*, thymidine kinase (MPN044); *thyA*, thymidylate synthase (MPN320); *tmk*, thymidylate kinase (MPN006); *adk*, adenylate kinase (MPN185); *gmk*, guanylate kinase (MPN246); *cmk*, cytidylate kinase (MPN476); *nrdE/nrdF*, ribonucleotide reductase (MPN322 and MPN324); *pyrH*, uridylate kinase (MPN632); deoxyadenosine kinase (MPN386). I = inhibition.

*Mycoplasmas* differ from their hosts in the biosynthesis of precursors for DNA and RNA because they cannot synthesize purine and pyrimidine bases *de novo*. Therefore, they rely totally on the salvage pathway for nucleotide biosynthesis (depicted in Figure 4). Purine bases such as Hx, Gua, and Ade are recycled by HPRT and adenine phosphoribosyl transferase, whereas the pyrimidine base, uracil is salvaged by uracil phosphoribosyl transferase [31,32]. The salvage of deoxynucleosides is catalyzed by deoxynucleoside kinases, including TK and deoxyadenosine/deoxyguanosine kinase [29]. The deoxynucleoside monophosphates are further phosphorylated to their corresponding triphosphates and are used as precursors for DNA synthesis. The ribonucleoside monophosphates are further phosphorylated to their triphosphate forms, and are then incorporated into RNA, or the diphosphate forms can be reduced by ribonucleotide reductase to produce precursors for DNA synthesis (Figure 4). Of 17 genes involved in nucleotide biosynthesis, 15 are essential [33,34]. Therefore, it has been suggested that this pathway may be a therapeutic target for future development of antibiotics [42].

Our screening of 30 FDA-approved anticancer and antiviral nucleoside analogs revealed seven potent inhibitors of Mpn growth with MIC values at clinically achievable plasma concentrations. Nucleoside and nucleobase analogs used in anticancer and antiviral therapy are prodrugs. In order to exert their therapeutic potential they have to compete with natural substrates for uptake (e.g. transport across plasma membrane) and metabolism (e.g. enzymes that activate them to their active forms). Once phosphorylated these analogs are trapped inside the cells and further metabolized to their active form by cellular enzymes, therefore, competition/inhibition of enzymes (e.g. TK or HPRT) in the initial phosphorylation step would also affect the uptake and metabolism of these compounds, and thus their cytotoxic effect (Figure 4). As shown in Table 2, dipyridamole and 6-TG inhibited Hx and Gua uptake and metabolism but not Ade or Ura, suggesting that HPRT may be an immediate target. Pyrimidine nucleoside analogs e.g. TFT, 5FdU and dFdC affected the uptake and metabolism of all radiolabeled substrates, indicating that multiple enzymes/steps are involved and the extent of uptake of specific nucleoside or nucleobase is determined by activities of these metabolic enzymes (Figure 4).

The 6-TG inhibited Mpn growth with MIC value of 0.20 μg ml^-1^, which is equivalent to tetracycline (MIC = 0.1 μg ml^-1^). However, 6-MP, a 6-TG analog did not inhibit Mpn growth. Neither theophylline, 7-(2, 3-dihydroxypropyl) theophylline, allopurinol, nor caffeine inhibited Mpn growth. 6-TG strongly inhibited uptake and incorporation of nucleotides derived from Hx and Gua into DNA and RNA, indicating that the observed inhibition by 6-TG was both at the level of transport and metabolism. It is noteworthy that the uptake/metabolism of Hx and Gua was inhibited by all the analogs used.

Thiopurines, especially mercaptopurines, are the first line drugs for the treatment of acute leukemia since the 1950s. They are also used in the treatment of inflammatory bowel disease [43]. The 6-TG and 6-MP exert their cytotoxicity through incorporation into DNA as deoxy-6-thioguanosine. These thiopurines are metabolized to deoxy-6-thioguanosine triphosphate via the purine salvage pathway initiated by HPRT (Figure 4). Thiopurine methyl transferase is a key enzyme in converting mercaptopurine to its cytotoxic metabolites, which can either inhibit purine nucleotide biosynthesis or incorporate into DNA or RNA, causing DNA damage and cell death [37]. Mpn does not possess the essential enzymes, inosine monophosphate dehydrogenase and thiopurine methyl transferase, to convert mercaptopurine to the cytotoxic thioguanine nucleotides, the respective methyl thiopurine nucleotides. This may explain why 6-MP did not inhibit Mpn growth.

To further investigate the mechanism by which 6-TG inhibited Mpn growth, Mpn HPRT was expressed, purified, and characterized. Both Hx and Gua are good substrates for the enzyme and the V_max_ values for these substrates are in the same order of magnitude as the human enzyme [44]. In humans, the plasma concentrations of Hx and Gua are approximately 172 μM and 97 μM [45], which is close to the K_m_ and S_0.5_ values of Mpn HPRT with Hx and Gua. These results suggest that Mpn HPRT is capable of efficiently salvaging both Hx and Gua. In addition, Mpn HPRT showed positive cooperativity with Gua, indicating that at higher Gua concentration the enzyme utilizes Gua better.

6-TG and 6-MP are structural analogs. The observed significant differences in their inhibitory effects with Mpn and human HPRT suggest that there are structural differences in binding of these two compounds to the respective HPRTs in their active sites. These differences could be used in future design of *Mycoplasma* specific inhibitors. HPRT has been suggested as a target for anti-parasite drug development and new compounds have been developed [46].

Halogenated pyrimidine analogs such as 5FdU inhibited Mpn and *Ureaplasma* growth, as reported in our earlier studies [30,35]. Others have shown that TFT and 5FdU inhibited *Cryptosporidium* growth [47], and gemcitabine inhibited *Staphylococcus aureus* growth *in vitro* and in animal models [48]. In this study, several halogenated pyrimidine analogs inhibited Mpn growth, and TFT and dFdC were more potent than 5FdU. The mechanism of inhibition by dFdC is most likely due to inhibition of ribonucleotide reductase and incorporation into DNA by dFdC metabolites (Figure 4). We did not observe significant differences in the inhibitory effects between the wild type and the *thyA* mutant strains, suggesting that TS activity is not required for toxicity of these compounds to Mpn.

*Mycoplasma* TK is an essential enzyme while TS is not [31,33,34]. The expression of TK in Mpn was correlated with Mpn growth and DNA synthesis, and upregulation of TK activity was observed in an Mpn strain lacking TS activity [31]. The phosphorylated products of TFT and 5FdU by TK irreversibly inhibit TS activity via covalent binding to the enzyme, and down regulation of TS activity leads to upregulation of TK activity, similar to what was observed with the *thyA* mutant [31]. Increased salvage of dT due to the induction of TK activity leads to higher level of dTTP, an allosteric regulator of purine nucleotide reduction by ribonucleotide reductase. Inhibition of ribonucleotide reductase activity by high level of dTTP led to decreased uptake and incorporation of labelled nucleobases as shown in this study, which may result in imbalance in nucleotide pools. In addition, high TK activity facilitates the phosphorylation of TFT and 5FdU and accumulation of TFT-TP and 5FdUTP that may affect the integrity of DNA and lead eventually to cell death (Figure 4). The fact that both TFT and 5FdU inhibited the growth of both wild type and the *thyA* mutant strain to the same extent, and the TK activity is upregulated by TFT and 5FdU, suggests that TK plays an important role in growth inhibition observed with these compounds.

## Conclusions

In this study we have shown that several anticancer and antiviral nucleoside and nucleobase analogs are potent inhibitors of Mpn growth and that the plausible mechanism of growth inhibition by these analogs are due to inhibition of enzymes in the nucleotide biosynthesis pathway and nucleoside transporter. We should keep in mind that the analogs used in this study are potent anticancer and antiviral drugs and most of them have diverse adverse side effect in humans and therefore, they may not be suitable for treatment of a mild Mpn infection. However, the results obtained with these analogs may be used as leads in the design of *Mycoplasma* specific inhibitors, substrates, or non-substrate inhibitors for the target enzymes in order to reduce the risk of host cell toxicity. More work regarding the mechanism of action of these drugs is needed. This study has provided the basis for future development of antibiotics against *Mycoplasma* or other bacteria.

## Methods

### Materials

Radiolabelled substances: [^3^H]-hypoxanthine ([^3^H]-Hx, 13.8 Ci/mmol), [^3^H]-thymidine ([^3^H]-dT, 20 Ci/mmol), [γ-^32^P]-ATP (2000 Ci/mmol), [^3^H]-Uracil ([^3^H]-Ura, 31.65 Ci/mmol), and [^3^H]-adenine ([^3^H]-Ade, 27.2 Ci/mmol) were purchased from PerkinElmer. [^3^H]-guanine ([^3^H]-Gua, 10.7 Ci/mmol) and [5-^3^H]-deoxyuridine 5’-monophosphate ([^3^H]-dUMP, 27 Ci/mmol) were from Moravek Biochemicals, Inc. The nucleoside and nucleobase analogs library [36] was kindly provided by Professor Pär Nordlund, from the Karolinska Institute, Stockholm, Sweden. Phosphoribosyl pyrophosphate (PRPP), dipyridamole, tetracycline, and nonradioactive Hx and Gua were from Sigma-Aldrich.

### Mpn culture, and the effects of nucleoside and nucleobase analogs on growth and metabolism

Nucleoside and nucleobase analogs were dissolved in dimethyl sulfoxide (DMSO) as stock solutions and diluted with Mpn culture medium to the desired concentration immediately prior to use. The DMSO concentration in the final dilution was < 1%, which would not interfere with Mpn growth.

Mpn laboratory strain M129 wild type and a *thyA* mutant strain [31] were used in this study. Mpn was cultured at 37°C in a CO_2_ incubator using 75 cm^2^ tissue culture flasks containing 50 ml Hayflick’s medium, and harvested at day 4 when the medium color change was observed [49]. The cells were harvested and the pellet was resuspended in 6 ml fresh medium and the cfu/ml was determined by serial dilution (10-fold) and plating on broth agar plate. Colonies was counted and cfu/ml was calculated.

Inhibition studies were performed in 96-well plates containing 200 μl Mpn culture (approximately 10^6^ cfu ml^-1^) in Hayflick’s medium and 200 μl each compound in series dilutions (2-fold) with the growth medium, with three to four replicas. The plates were sealed with clear adhesive sheets and incubated at 37°C incubator. Absorbance ratio at 450 nm and 560 nm was used as Mpn growth index, which was measured daily, and by visual detection for at least 8 days, as previously described [32]. In the absence of inhibitor, the culture medium turned yellow on day 4. Controls were cultured in the presence of 2 μg/ml tetracycline, which showed no growth for up to 8 days. Medium was placed in four wells per plate for controls, which showed no color change during the incubation period. The MICs (minimal inhibitory concentration required to inhibit Mpn growth to 90%) were determined as the lowest concentration at which the growth index was ≈ 10% of the control values (at the time when the control culture medium color turned yellow), essentially as described [50].

Nucleoside and nucleobase uptake and metabolism was done with the wild type strain, which was cultured in 25 cm^2^ tissue culture flasks, inoculated with 1 ml stock culture (1 × 10^8^ cfu/ml) Mpn, in the presence of tritium labeled dT, Hx, Gua, Ade or Ura (1 μCi ml^-1^) and the presence or absence of nucleoside and nucleobase analogs (10 μM) and incubated at 37°C for 70 hours. The cells were harvested and analyzed essentially as described [31]. Analogs used were dipyridamole, 6-thioguanine (6-TG), trifluorothymidine (TFT), gemcitabine (dFdC) and 5-fluorodeoxyuridine (5FdU). Total uptake is the percent of radioactivity recovered in the cells divided by total radioactivity added to the growth medium. Percent of acid insoluble (radioactivity found in DNA and RNA) was also calculated [31]. These experiments were done more than three times and data are given as mean ± SD.

To determine the effect of TFT on TK and TS activity, Mpn wild type cells were cultured in 75 cm^2^ tissue culture flasks containing 50 ml medium, inoculated with 3 ml of stock culture (1 × 10^9^ cfu/ml), in the presence of [^3^H]-dT (1 μCi ml^-1^) and different concentrations of TFT. After 70 hours at 37°C the cultures were harvested and divided to two aliquots, one was used to determine total uptake/metabolism of radiolabeled dT and total proteins were extracted from the other aliquot and used to measure TK and TS activity using [^3^H]-dT and [5-^3^H]-dUMP as substrates [31].

### Expression and purification of recombinant Mpn HPRT

The Mpn HPRT gene (MPN672) coding sequence was codon optimized for expression of the recombinant protein in *E. coli,* by using the Proprietary OptimumGene™ codon optimization technology combined with gene synthesis (GenScript Inc.), and the synthetic cDNA was then cloned into the pEXP5NT vector (Invitrogen), and expressed as an N-terminal fusion protein with a 6xHis tag and a TEV cleavage site. The plasmid containing the MPN672 gene was then transformed into the BL21 (DE3) pLysS strain and the recombinant protein production was induced by addition of 0.1 mM IPTG at 37°C for 4 h. The cells were harvested by centrifugation at 2000 × *g* for 25 min at 4°C. The pellets were resuspended in lysis buffer containing 25 mM Tris/HCl, pH 7.5, 2 mM MgCl_2_, and 0.4 M NaCl. The cells were lysed by repeated freezing and thawing, and sonication for 2 min in an ice/water bath. After centrifugation at 25,000 × *g* for 30 min at 4°C, the supernatant was used to purify the recombinant protein by metal affinity chromatography on a Ni-Sepharose (GE Healthcare) resin column, and the Mpn HPRT was eluted with 0.4 M imidazole in lysis buffer. The eluted fractions were analyzed by 12% SDS-PAGE and those containing purified enzyme were pooled and passed through a PD-10 column (GE Healthcare) for desalting and buffer exchange. The final enzyme preparation was in a buffer containing 10 mM Tris/HCl, pH 7.5, 5 mM MgCl_2_, 1 mM dithiothreitol (DTT), and 20% glycerol, and stored in aliquots at −70°C. Protein concentration was determined by Bio-Rad protein assay using bovine serum albumin (BSA) as a standard.

Recombinant human TK1, human TK2, *Ureaplasma* TK, and human HPRT were expressed and purified as previously described [30,40,44,51].

### Enzyme assays

The HPRT assay was performed by using the DE-81 filter paper assay with tritium labeled hypoxanthine ([^3^H]-Hx) or guanine ([^3^H]-Gua) as substrates, essentially as previously described [44]. Briefly, the reaction mixture contained 50 mM Tris/HCl, pH 7.5, 10 mM MgCl_2_, 5 mM DTT, 15 mM NaF, 1 mM PRPP, and various concentrations of [^3^H]-Hx or [^3^H]-Gua in a total of 50 μl. The reaction was initiated by addition of the enzyme, and at 0, 5, 10, and 15 min intervals, 10 μl reaction mixture was withdrawn and spotted onto the DE81 filter paper and dried. The unreacted substrate was washed and the products were eluted and counted in a liquid scintillation counter. With [^3^H]-Gua as substrate the reaction (in a total of 25 μl) was initiated by addition of the enzyme (10 μl), incubated at 37°C for 2 min, stopped by addition of 1 M HCl (10 μl), and placed immediately on ice. After neutralization, 15 μl of the mixture was spotted onto the DE81-filter paper. The filters were then washed, and the products were eluted and counted by liquid scintillation.

IC_50_ values for purine analogs were determined for both Mpn HPRT and human HPRT using fixed concentrations of [^3^H]-Hx (10 μM) or [^3^H]-Gua (10 μM) and variable concentrations of the inhibitors.

Thymidine kinase assay was performed using tritium labelled thymidine ([^3^H]-dT) as substrate and various concentrations of the inhibitors essentially as previously described [40] to determine the IC_50_ values of TFT and 5FdU.

Kinetic parameters for TFT were determined by using the phosphoryl transfer assays as previously described [52]. Briefly, each reaction was performed in a total volume of 20 μl containing 50 mM Tris/HCl, pH 7.5, 0.5 mg/ml BSA, 5 mM DTT, 2 mM MgCl_2_, 15 mM NaF, variable concentrations of TFT, 0.1 mM [γ-^32^P]-ATP, and 50 ng purified enzyme at 37°C for 20 min, and stopped by heating at 70°C for 2 min. After brief centrifugation, 1 μl supernatant was spotted onto a TLC plate (PEI-cellulose, Merck) and dried. The TLC plates were developed in isobutyric acid/ammonia/H_2_O (66:1:33). The reaction products were visualized and quantified by phosphoimaging analysis (Quantity One, Bio-Rad).

### Statistical analysis

The data were analysed by unpaired student’s *t*-test (two tailed) using GraphPad Prism 5 software. P < 0.05 is considered as significant.

## Competing interests

Both authors declare that they have no competing interests.

## Authors’ contributions

RS performed the kinetic and inhibitions studies with thymidine kinases, analyzed the data and created the figures; LW designed the study, performed growth inhibition studies, uptake and metabolism of labelled nucleosides, characterized Mpn HPRT; analyzed the data and wrote the manuscript. All authors have read and approved the manuscript.
